# Humoral Immune Response to SARS-CoV-2 Vaccination after a Booster Vaccine Dose in Two Kidney Transplant Recipients with Fabry Disease and Variable Secondary Immunosuppressive Regimens

**DOI:** 10.3390/vaccines9121412

**Published:** 2021-11-30

**Authors:** Lavinia Bernea, Oana R. Ailioaie, Nadine Benhamouda, Eric Tartour, Dominique P. Germain

**Affiliations:** 1 French Referral Centre for Fabry Disease, Division of Medical Genetics, APHP–Paris Saclay University, 92380 Garches, France; lavinia-maria.bernea@aphp.fr (L.B.); assistants.genetique.rpc@aphp.fr (O.R.A.); 2 Department of Immunology, INSERM U970, APHP Hôpital Européen Georges Pompidou and University of Paris, 75015 Paris, France; nadine.benhamouda@aphp.fr (N.B.); eric.tartour@aphp.fr (E.T.); 3 Second Department of Internal Medicine, First Faculty of Medicine, Charles University, 128 08 Prague, Czech Republic; 4 Division of Medical Genetics, University of Versailles, 78180 Montigny, France

**Keywords:** COVID-19, vaccine, Fabry disease, renal transplant

## Abstract

The urgent need to fight the COVID-19 pandemic has accelerated the development of vaccines against SARS-CoV-2 and approval processes. Initial analysis of two-dose regimens with mRNA vaccines reported up to 95% efficacy against the original strain of the SARS-CoV-2 virus. Challenges arose with the appearance of new strains of the virus, and reports that solid organ transplant recipients may have reduced vaccination success rates after a two-dose mRNA vaccination regimen encouraged health authorities to recommend a booster in immunocompromised patients. Fabry disease is an X-linked inherited lysosomal disorder, which may lead to chronic end-stage renal disease. We report on two patients with advanced Fabry disease, renal graft and adjunctive immunosuppressive therapies who exhibited variable humoral vaccination-related immune responses against SARS-CoV-2 after three vaccine doses. The first patient developed mild COVID-19 infection, while the second patient did not seroconvert after three shots of an mRNA vaccine. Both cases emphasize that patients with Fabry disease and renal graft are susceptible to develop a weak response to COVID-19 vaccination and highlight the importance of maintaining barrier protection measures. Vaccination of family members should be encouraged to lower the risk of viral transmission to immunocompromised, transplanted patients, including vaccinated ones.

## 1. Introduction

On 11 March 2020, the World Health Organization (WHO) declared the coronavirus disease 2019 (COVID-19) outbreak a pandemic, with over 249,743,428 cases and 50,474,652 deaths reported as of 8 November 2021 [1]. The rapid spread of coronavirus disease 2019 (COVID-19) caused by *severe acute respiratory syndrome coronavirus 2* (SARS-CoV-2) has prompted major research efforts for the development of new technologies aiming at reducing the mortality and morbidity rates of individuals infected by the SARS-CoV-2 virus.

The urgent need to lower the high contamination levels has shortened the usual timeframe in the clinical development programs of vaccines [2] and unlike historical pandemics, the fight against COVID-19 has benefited from the rapid development of vaccines, including several based on novel technologies. Beside traditional vaccines (based on attenuated or inactivated viruses), the world has witnessed the large-scale employment of replication-deficient vectors such as ChAdOx1-S [3] and Ad.26.COV2.S [4], as well as mRNA vaccines, which may also pave the way for future therapeutics advances [5]. The rapid emergence of novel mRNA technologies has left the scientific community and regulatory agencies with uncertainties and queries regarding the long-term efficacy and safety of those novel vaccinal approaches.

The BNT162b2 mRNA vaccine (COMIRNATY^®^, Pfizer, New York, NY, USA) is a lipid nanoparticle-formulated, nucleoside-modified RNA, coding for the full-length spike protein of the SARS-CoV-2 virus. An interim analysis of the preliminary phase 3 clinical trial, with a two-dose regimen (21 days apart), reported a 95% efficacy against the original strain of the SARS-CoV-2 virus [6]. A similar technology is used in the mRNA-1273 vaccine (SPIKEVAX^®^, Moderna, Cambridge, MA, USA) with a reported 94.1% efficacy in the interim analysis of the phase 3 COVE clinical trial [7].

The evolution of the pandemic was marked by the surge of new strains of the SARS-CoV-2 virus, which questioned the efficacy of the available vaccines against the four variants of interest: the alpha variant (B.1.1.7), the beta variant (B.1.351), the gamma variant (P.1) and the delta variant (B.1.617.2). A recent analysis on vaccine efficacy against the latest, currently predominantly circulating B.1.617.2 (delta) variant showed lower effectiveness of a two-dose regimen of both the BNT162b2 and ChAdOx1nCoV-19 vaccines [8].

Moreover, preliminary data have suggested that in specific subpopulations, two doses of the mRNA vaccines may not always be able to confer complete serological immune response in immune-compromised patients such as solid organ transplant recipients [9,10]. Whether cellular immunity might bring additional protection to the humoral immunity is unknown and its exact potential benefits remain difficult to assess and quantify and warrant further studies [11].

Fabry disease (FD; OMIM #301500) is a rare, X-linked inherited lysosomal disorder caused by pathogenic variants in the gene encoding α-galactosidase (*GLA*) [12]. Deficient α-galactosidase enzyme activity leads to progressive storage of globotriaosylceramide (Gb_3_) and its deacylated derivative, globotriaosylsphingosine (lysoGb_3_), resulting in a cascade of pathogenic effects, with cardiovascular [13], cerebrovascular [14] and renal complications [15]. When the α-galactosidase activity is severely deficient, FD may lead to chronic end-stage renal disease in male patients and females with unfavourable X chromosome inactivation profiles [16], ultimately requiring renal grafts with adjunctive immunosuppressive therapies [17].

Whether the current proposed recommendations to administer a third, additional vaccine dose to recipients of solid organ grafts also applies to patients with Fabry disease has not been studied yet [18].

Here, we report the preliminary experience from a tertiary referral centre for Fabry disease through two cases of patients with FD and kidney transplants who developed variable humoral responses against SARS-CoV-2 after booster vaccine doses.

### 1.1. Case Report #1

A 60-year-old male patient, who had received a renal graft in 1992 for end-stage renal disease of unknown aetiology at the age of 31, was diagnosed with Fabry disease one year later with the identification of a c.59_73delCCCTCGTTTCCTGGG (p.Ala20_Trp24del) pathogenic variant in exon 1 of the *GLA* gene. Enzyme replacement therapy (ERT) with agalsidase beta (1 mg/kg every other week (EOW)) was initiated in 2001 and continued for eight years, followed by a switch to agalsidase alfa (0.2 mg/kg EOW) in 2009, in the context of a worldwide shortage of agalsidase beta. In 2013, the patient was switched back to agalsidase beta, on which he has remained ever since, at the recommended dose of 1 mg/kg EOW.

The patient had experienced several severe cardiac clinical events, with multiple ischemic events and a myocardial infarction before initiation of ERT. He subsequently required active stents on the anterior interventricular artery (2012) and the right coronary artery (2013). The patient also suffered from a vascular stroke in 2005 and three transient ischemic attacks in 2011. Other FD manifestations include angiokeratoma, acroparesthesia, hypohidrosis and bilateral tinnitus.

The patient’s glomerular filtration rate (eGFR) estimated with the Chronic Kidney Disease EPIdemiology Collaboration (CKD-EPI) formula was 50 mL/min/1.73 m^2^ in year 2021 (almost thirty years after kidney transplantation), while on maintenance immunosuppression therapy with ciclosporin A 75 mg twice a day (BID). He had neither proteinuria nor tubular dysfunction.

The patient received anti-COVID 19 vaccination prophylaxis, with a first shot of BNT162b2 mRNA (COMIRNATY^®^) administered on 26 February 2021 (EP2163 batch), and a second shot on 26 March 2021 (ER9470 batch). His serological response assayed after the second dose of the vaccine was positive for anti-S antibodies: >250 UI/mL (normal values < 0.8 UI/mL). In France, renal graft recipients are encouraged to adopt a three-dose vaccination scheme on the basis of published reports that immunosuppressive regimens may interfere with their immune responses [10], so despite satisfactory serological response, the patient was offered a third shot of BNT162b2 mRNA, which he accepted and received on 28 April 2021 (ET6956 batch). A second serology assay, carried out on 12 May 2021, showed a major increase in the IgG titres to >2080 binding antibody units (BAU)/mL (positivity > 33.8 BAU/mL).

On 13 July 2021, the patient experienced fatigue and generalized myalgia, together with odynophagia, rhinorrhoea and occasional dry cough. He described an isolated episode of diarrhoea without fever. On 27 July, he complained of anosmia and ageusia. His wife, who had completed a two-dose BNT162b2 mRNA anti-COVID-19 vaccination scheme, had experienced similar symptoms one week earlier, while their unvaccinated 16-year-old daughter described COVID-19 symptoms 4 days thereafter. A PCR test for the SARS-CoV-2 virus carried out on 20 July 2021 confirmed the patient’s infection with the delta strain (p.L452R variant) of the SARS-CoV-2 virus. A chest computer tomography (CT) performed on 22 July 2021 did not show any pulmonary sign of severe COVID-19 involvement. Laboratory parameters revealed a mild inflammatory syndrome with CRP slightly increased at 6.4 mg/L (normal values < 5 mg/L), without leukocytosis. D-dimers were in the normal range (285 ng/mL), and liver enzymes were not elevated. Cardiac markers were stable, with troponin I at 18.1 ng/L (normal value < 34 ng/L) and BNP at 111 pg/mL (versus 150 pg/mL prior to the SARS-CoV-2 infection, with a normal value < 100 pg/mL). The patient’s renal function remained stable (CKD-EPI eGFR: 51 mL/min/1.73 m^2^), with no proteinuria. The patient did not require hospitalisation and was managed at home. The COVID-19 resolved without sequelae.

Patient #1’s cellular immunity to SARS-CoV-2 was subsequently studied on 2 November 2021, six months after the booster vaccine dose and three months after the mildly symptomatic COVID-19. A modest but significant anti-spike CD4^+^ T helper cells response was detected. No CD8^+^ TL response to peptides derived from the S1 and S2 domains of the spike protein could be detected using the IFNγ Elispot technique (Table 1).

### 1.2. Case Report #2

A 62-year-old patient was diagnosed with end-stage renal disease in 1998 at the age of 39 years, prompting a renal biopsy which revealed a histological pattern suggestive of Fabry disease. Genotyping disclosed a c.640-3C > G pathogenic splice-site variant in intron 4 of the *GLA* gene.

Progressive deterioration of the patient’s renal function led to a renal graft in October 2000. After the renal transplantation, a vascular rejection of the transplant was treated by plasmapheresis, OKT3 and pulse corticosteroids with a favourable outcome within one month.

From October 2002 to October 2010, the patient received ERT with agalsidase beta (1 mg/kg EOW), before switching to agalsidase alfa (0.2 mg/kg EOW) in 2010. Despite ERT and concomitant treatments, FD continued to progress. The patient experienced a vertebrobasilar cerebrovascular accident in 2011. His cardiac function also worsened, with left ventricle hypertrophy and conduction defect. This led to a reverse switch of the ERT to agalsidase beta (1 mg/kg EOW) in 2012 and the patient has remained on the latter preparation ever since.

The patient’s renal function was recently estimated at 40 mL/min/1.72 m^2^ (CKD-EPI) while on maintenance immunosuppression therapy with tacrolimus 0.5 mg BID and mycophenolate mofetil 500 mg QD. His neurological involvement has remained globally stable, while a non-ST elevation myocardial infarction (NSTEMI) occurred in February 2020, needing an active stent, together with cryoablation of paroxystic atrial fibrillation, in March 2020. The last MRI quantification of the interventricular septum thickness was 17 mm.

The patient initially received a two-dose vaccinal scheme, with a first shot of BNT162b2 mRNA (COMIRNATY^®^) on 21 January 2021 (EP9598 batch) and a second injection on 18 February 2021. His serological response assayed three months after the second dose was negative for anti-S antibodies, with a value of <7.8 AU/mL (positivity limit > 49 AU/mL). On the basis of the absence of seroconversion, a third dose of the BNT162b2 mRNA vaccine was administered on 25 May 2021 (FC0681 batch). A second serology, performed on 21 June 2021 after the three-dose scheme, was still negative, with anti-S protein IgG titres at 24 AU/mL. Cellular immunity could not be explored.

## 2. Discussion

We report on two patients with advanced Fabry disease, renal graft and maintenance immunosuppression treatment who developed unexpected clinical outcomes following SARS-CoV-2 booster vaccine doses.

The first patient developed COVID-19 despite a three-dose mRNA vaccination scheme. The patient had a modest positive serological response after his second dose of the BNT162b2 vaccine. His anti-S antibodies titres significantly increased after receiving a third dose of the BNT162b2 mRNA vaccine. In spite of his humoral protection, he contracted the delta strain of the SARS-CoV-2 virus. However, although on immunosuppressive therapy (ciclosporine 75 mg BID resulting in a C0 = 168.7 ng/mL), he only developed a mild form of COVID-19, with neither pulmonary involvement [19] nor any organ failure. This emphasizes the importance of vaccination to decrease the disease burden and morbidity of COVID-19 in patients with genetic disease and renal grafts submitted to a secondary immunosuppressive regimen, such as advanced FD patients. Despite his immunosuppressive treatment, anti-SARS-CoV-2 CD4^+^ T cells could be detected in this patient without being able to discriminate the respective role of vaccination or SARS-CoV-2 infection in this induction. In contrast, no anti-SARS-CoV-2 CD8^+^ T cells could be detected in the patient, which is consistent with literature data on the lower induction of those cells compared to CD4^+^ T cells [20].

The second case was a 60-year-old male patient, with advanced Fabry disease and immunosuppressive therapy for his renal graft. He did not seroconvert after a two-dose vaccinal scheme and remained seronegative after an additional, third shot of BNT162b2.

Reports that recipients of solid organ transplants may develop a weak humoral response after a two-dose mRNA vaccination regimen, with only 15% serological conversion after a first dose and 54% conversion after the full scheme, [10] have led the French health authorities to recommend a third shot for immunocompromised patients. Evidence of higher seroconversion after a three-dose formula has been documented with a reported increase in serological response from 40% to 68% after the second and third doses, respectively, and no reports of COVID-19 in a cohort of transplanted patients (*n* = 101), among which 78 had received kidney grafts [21]. Another report on 30 transplanted patients (26 kidney graft recipients) showed increasing titres for individuals who had previously responded after the second dose, such as our first patient, but with persistent absence of seroconversion in 16 patients (67%) after administration of the third dose [22]. Kidney transplantation was found to be an independent risk factor for negative SARS-CoV-2 anti-S IgG antibodies response, 28 days after a second vaccine injection in a cohort of transplanted patients [23]. Of note, among patients with solid organ transplantation, there might be differences in the serological response according to the classes and doses of the immunosuppressive agents. Belatacept, mycophenolate mofetil (MMF), mycophenolic acid (MPA) and calcineurin inhibitors have a significant influence on the humoral response, while the influence of glucocorticosteroids and mTOR inhibitors is much weaker [24,25]. Patient #2, who did not seroconvert, was on mycophenolate mofetil, the ester prodrug of MPA, as part of his maintenance immunosuppressive treatment.

This is, to the best of our knowledge, the first report of COVID-19 following administration of a three-dose vaccination scheme, although it should be noted that the symptoms remained mild in this PCR-proven SARS-CoV-2 infection with the delta variant in a kidney transplant recipient with a secondary immunosuppressive regimen. Whether a change in the vaccine brand used as a booster would have resulted in full protection against COVID-19 could not be evaluated, since both patients received three doses of the BNT162b2 vaccine. Two different timings were employed: an interval of 4 weeks separated the second and third doses in the first patient while the second patient received his third dose more than three months after the second one. Whether an earlier administration of the third dose would have had a more favourable outcome on seroconversion in Patient #2 is unknown.

It should be noted that the second patient was never infected with COVID-19, although no data is available on his exposure to the SARS-CoV-2 virus. Cellular immunity has been shown to develop following vaccination against SARS-CoV-2, but there are limited data in the literature on its protective role. A limitation of our study is that cellular immunity was assessed only after SARS-CoV-2 infection in the first patient and could not be explored in the second patient. It is currently unknown whether the three-dose vaccination scheme may have conferred protection through cellular immunity thereby contributing to the absence of SARS-CoV-2 infection in Patient #2. The results of a prospective, multicenter observational study investigating the efficacy of COVID-19 vaccines in dialysis and kidney transplant patients and assessing both humoral and cellular responses were recently published [25]. SARS-CoV-2-vaccination induced seroconversion efficacy was markedly impaired in kidney transplant recipients (42%). T-cellular immunity largely mimicked humoral results. Major risk factors of seroconversion failure were immunosuppressive drug number and type (belatacept, MMF-MPA and calcineurin inhibitors) as well as vaccine type (BNT162b2 mRNA). Seroconversion rates induced by mRNA-1273 compared to BNT162b2 were 49% to 26% in transplant patients, respectively. Specific IgG antibodies directed against the new binding domain of the spike protein (RDB) were also significantly higher in dialysis patients vaccinated by mRNA-1273 (95%) compared to BNT162b2 (85%, *p* < 0.001). Vaccination appeared safe and highly effective, demonstrating an almost complete lack of symptomatic COVID-19 disease after boost vaccination [25].

Based on the incomplete serological response after a third dose in a percentage of immunocompromised patients, the possibility of a fourth dose has been proposed. However, the lack of clinical trials assessing the safety of four injections warrants further research.

Finally, both cases highlight the importance for vulnerable patients of a booster vaccine dose [24] and of maintaining barrier protection measures including (i) social distancing (ii) facial masks and (iii) frequent use of hydroalcoholic gel frictions [26] until the impact of immunosuppressive therapies on the immunogenicity and efficacy of SARS-CoV-2 vaccination in both cellular and humoral immune responses is better understood.

## Figures and Tables

**Table 1:** T cell immune responses explored after both booster vaccine dose and COVID-19 infection in Patient #1.

T Cell Immune Responses	Number of Spots	Positive Cut-Off (Spots/100,000 Cells)
CD4^+^ T cells		10
anti-S1	13	
anti-S2	14	
CD8^+^ T cells		
anti-S1	0	
anti-S2	1	

## Data Availability

The data presented in this study are available on request from the corresponding author. The data are not publicly available due to protection of patients’ privacy and confidentiality.
